# Supplementary material for: Consistent signatures in the human gut microbiome of old- and young-onset colorectal cancer
Source: Nat Commun. 2024 Apr 22;15:3396. doi: 10.1038/s41467-024-47523-x (PMC11035630; doi:10.1038/s41467-024-47523-x)
Supplement: Supplementary file 1 — Supplementary_information [file 41467_2024_47523_MOESM1_ESM.pdf]

Supplementary information

This supplementary information includes 11 supplementary figures and 1 supplementary table.

Supplementary Figure 1. Abundance distribution of taxa significantly differentiated in oCRC but not in yCRC.

Supplementary Figure 2. Abundance distribution of taxa significantly differentiated in yCRC but not in oCRC.

Supplementary Figure 3. Abundance distribution of the well-known CRC-enriched taxa *Bacteroides fragilis* and *Fusobacterium nucleatum* with potential carcinogenesis mechanism.

Supplementary Figure 4: Phylogenetic and genomic analysis of *Fusobacterium nucleatum* in CRC.

Supplementary Figure 5: Genomic analysis of *Fusobacterium animalis* in CRC.

Supplementary Figure 6: Phylogenetic and genomic analysis of *Bacteroides fragilis* in CRC.

Supplementary Figure 7: Phylogenetic and genomic analysis of *Escherichia coli* strain in CRC.

Supplementary Figure 8. Overall distribution of the Guangzhou samples based on microbial pathway profile.

Supplementary Figure 9. Overall distribution of the Guangzhou and Fudan samples based on microbial pathway profile.

Supplementary Figure 10. Abundance distribution of the well-known CRC-enriched microbial *cutC* gene.

Supplementary Figure 11. Prediction performance of microbial pathway-based classification models.

Supplementary Table 1. Prediction performance of microbial pathway-based classification models.

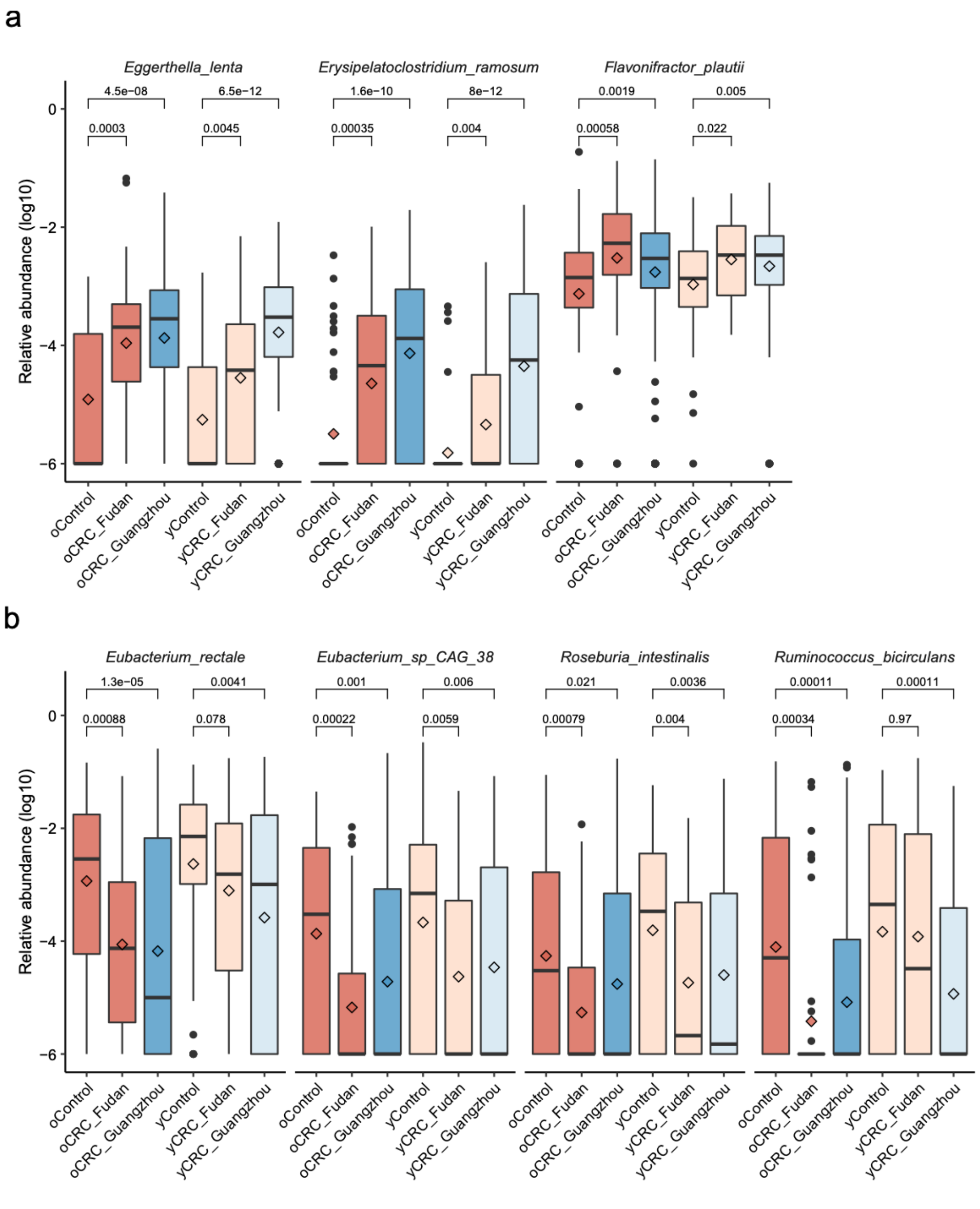

Supplementary Figure 1. Abundance distribution of taxa significantly differentiated in oCRC but not in yCRC. Top: Three microbial taxa enriched in oCRC groups at FDR adjusted P<0.05. These taxa were also increased in yCRC groups but with an FDR adjusted P value above 0.05. Bottom: Four microbial taxa depleted in oCRC groups (FDR adjusted P<0.05). These taxa were also depleted in yCRC groups but with an FDR adjusted P value above 0.05. P values on the top were calculated by two-side Wilcoxon rank-sum test (no FDR adjustment). The relative abundance is in log10 scale and zeros were replaced by a small value. Sample sizes for the compared groups: oControl (n=50), oCRC_Fudan (n=50), oCRC_Guangzhou (n=293), yControl (n=50), yCRC_Fudan (n=50), yCRC_Guangzhou (n=167). The boxplots show the median (thick line), interquartile range (box limits), 1.5× the interquartile range span (whiskers) and outliers (dots). Diamond shape indicates the mean abundance.

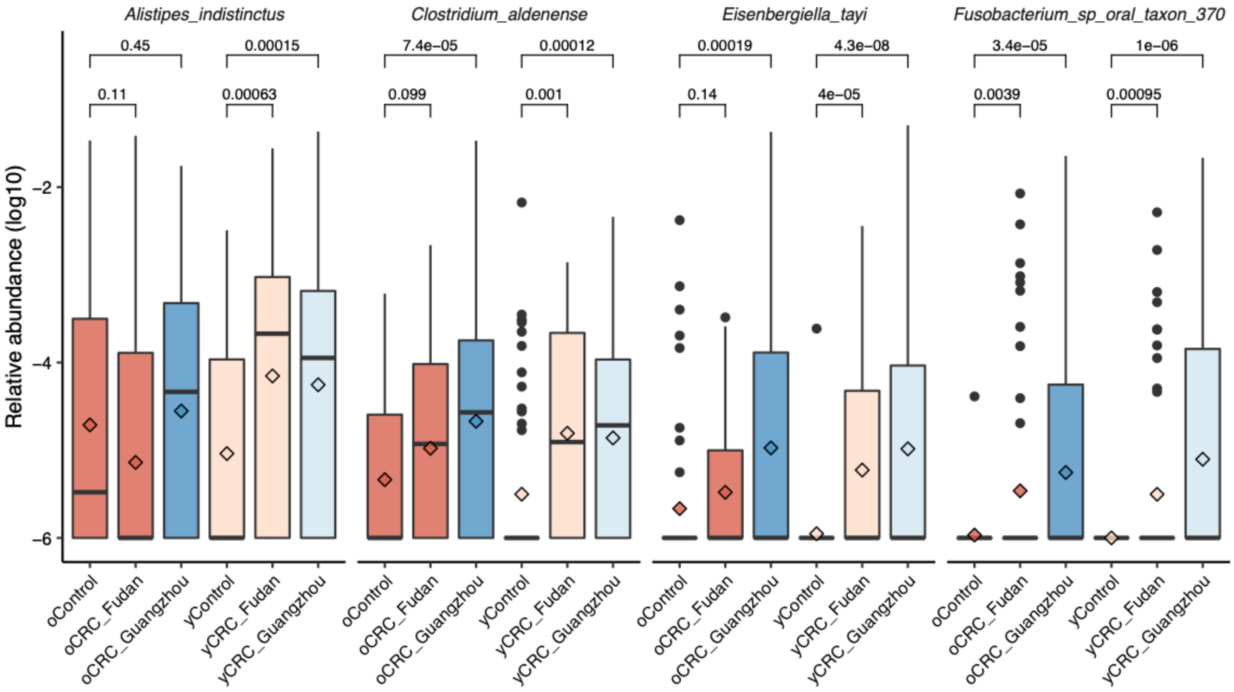

Supplementary Figure 2. Abundance distribution of taxa significantly differentiated in yCRC but not in oCRC. Four microbial taxa enriched in yCRC groups at FDR adjusted P<0.05. Three of them were also increased in oCRC groups but with an FDR adjusted P value above 0.05. P values on the top were calculated by two-side Wilcoxon rank-sum test (no FDR adjustment). The relative abundance is in log10 scale and zeros were replaced by a small value. Sample sizes for the compared groups: oControl (n=50), oCRC_Fudan (n=50), oCRC_Guangzhou (n=293), yControl (n=50), yCRC_Fudan (n=50), yCRC_Guangzhou (n=167). The meaning of boxplot is same to the description in **Supplementary Figure 1**.

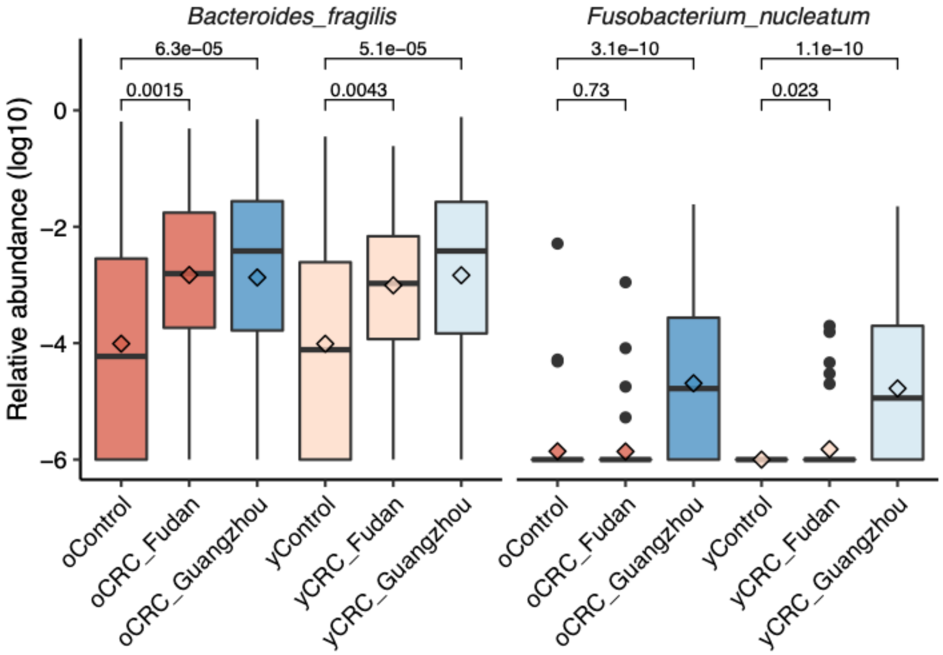

Supplementary Figure 3. Abundance distribution of the well-known CRC-enriched taxa *Bacteroides fragilis* and *Fusobacterium nucleatum* with potential carcinogenesis mechanism. The relative abundance is in log10 scale and zeros were replaced by a small value. P values on the top were calculated by two-side Wilcoxon rank-sum test (no FDR adjustment). Sample sizes for the compared groups: oControl (n=50), oCRC_Fudan (n=50), oCRC_Guangzhou (n=293), yControl (n=50), yCRC_Fudan (n=50), yCRC_Guangzhou (n=167). The meaning of boxplot is same to the description in **Supplementary Figure 1**.

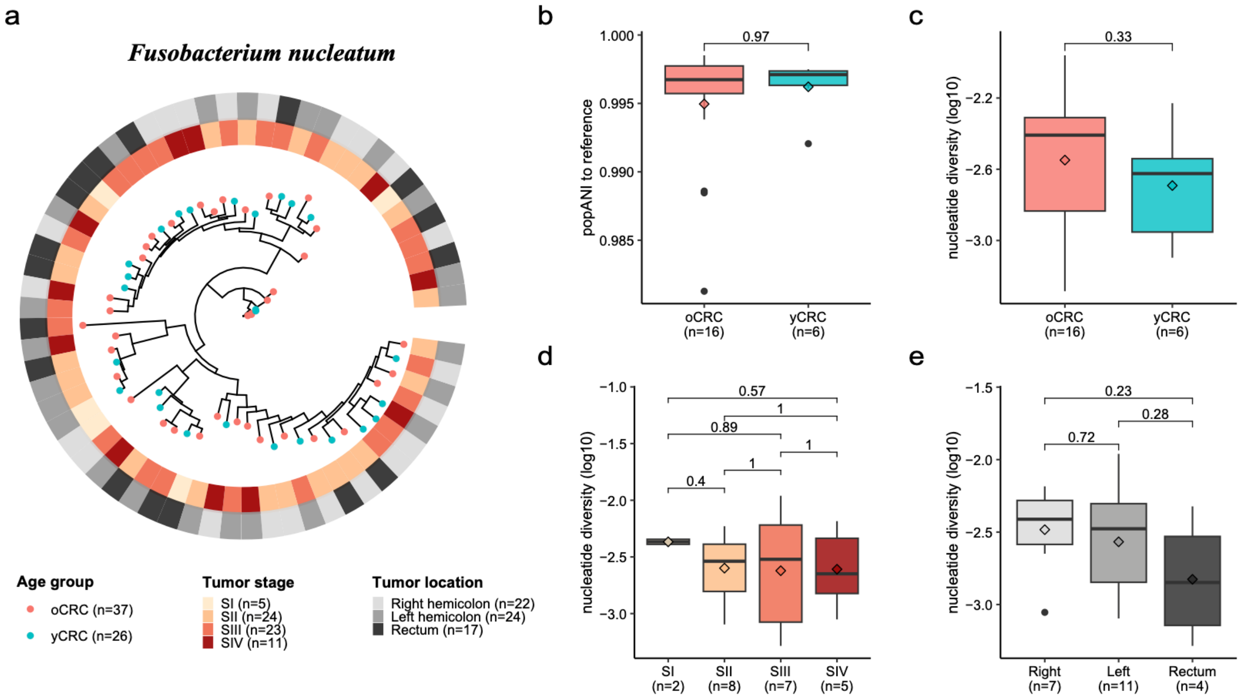

Supplementary Figure 4: Phylogenetic and genomic analysis of *Fusobacterium nucleatum* in CRC. (a) Phylogenetic tree of *F. nucleatum* constructed based on 36 marker genes. Tips are samples and colored by age group. Rings outside the tree indicate tumor stage and location. Only samples with reads mapped to at least 20 marker genes are displayed. (**b**) Population average nucleotide identity (popANI) values to reference genome (RefSeq GCF_008633215.1). (**c**), (**d**) and (**e**) are values of genome-wide nucleotide diversity stratified by age, tumor stage and location. Only panel (**a**) samples with reads mapped to the reference genome reaching a genome-wide breadth >0.1 and coverage >0.1 are shown. Genome breadth and coverage were determined using inStrain. Boxplot conventions are consistent with the description in **Supplementary Figure 1**.

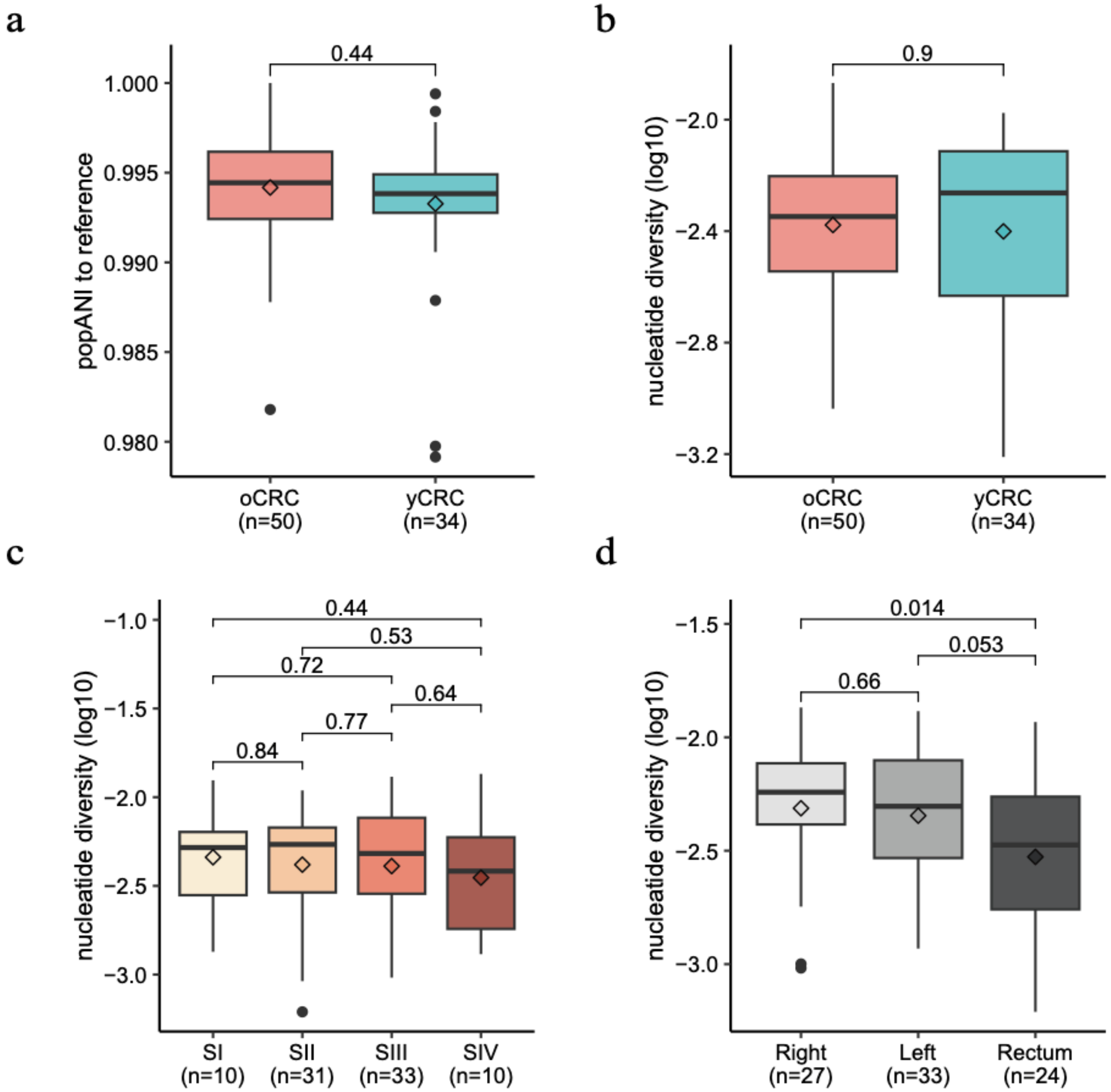

Supplementary Figure 5: Genomic analysis of *Fusobacterium animalis* in CRC. (**a**) Population average nucleotide identity (popANI) values to reference genome (RefSeq GCF_008633215.1). (**b**), (**c**) and (**d**) are values of genome-wide nucleotide diversity stratified by age, tumor stage and location. Only panel samples with reads mapped to the reference genome reaching a genome-wide breadth >0.1 and coverage >0.1 were shown. Boxplot conventions are consistent with the description in **Supplementary Figure 1**.

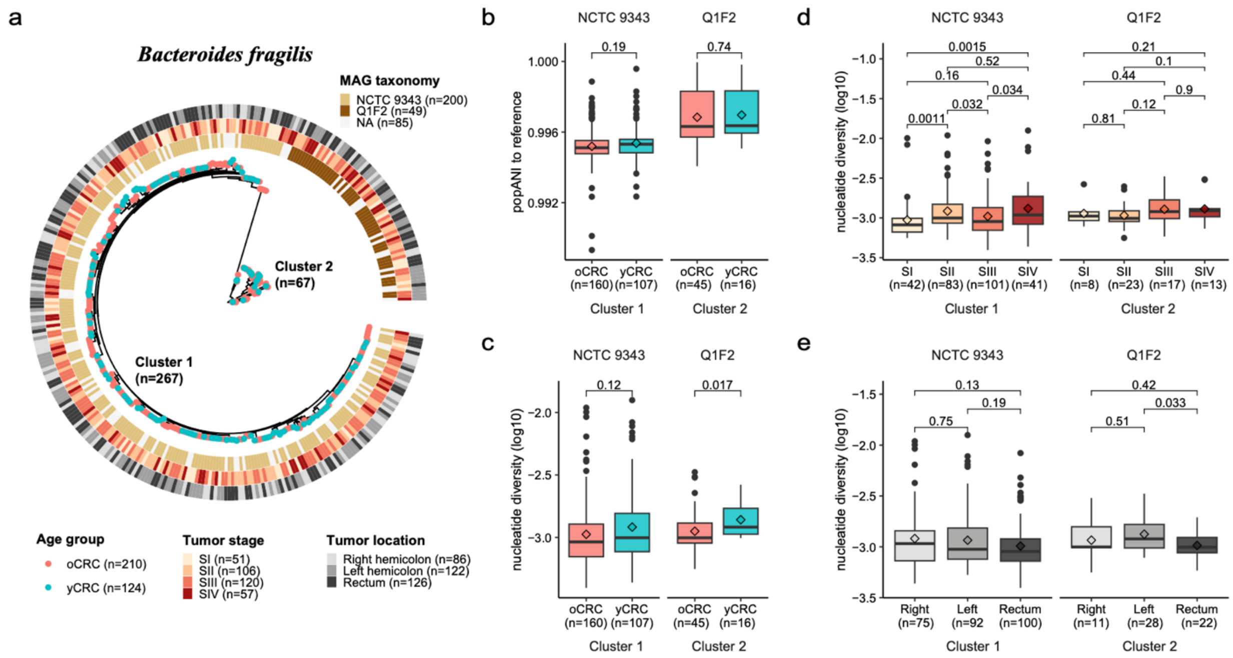

Supplementary Figure 6: Phylogenetic and genomic analysis of *Bacteroides fragilis* in CRC. (**a**) Phylogenetic tree of *B. fragilis* constructed based on 46 marker genes. Two distinct strain clusters are shown, and we named them as cluster 1 (dominated by samples with metagenome-assembled genomes (MAGs) annotated as strain NCTC 9343) and cluster 2 (dominated by samples with MAGs annotated as strain Q1F2). Tips are samples and colored by age group. Rings outside the tree indicate MAG taxonomy, tumor stage and location. Only samples with reads mapped to at least 20 marker genes are displayed. (**b**) Population average nucleotide identity (popANI) values to reference genomes of strain NCTC 9343 (RefSeq GCF_000025985.1) and Q1F2 (RefSeq GCF_002849695.1). (**c**), (**d**) and (**e**) are values of genome-wide nucleotide diversity stratified by strain cluster, age, tumor stage and location. Only panel (**a**) samples with reads mapped to the reference genome reaching a genome-wide breadth >0.5 and coverage >1 were shown. Boxplot conventions are consistent with the description in **Supplementary Figure 1**.

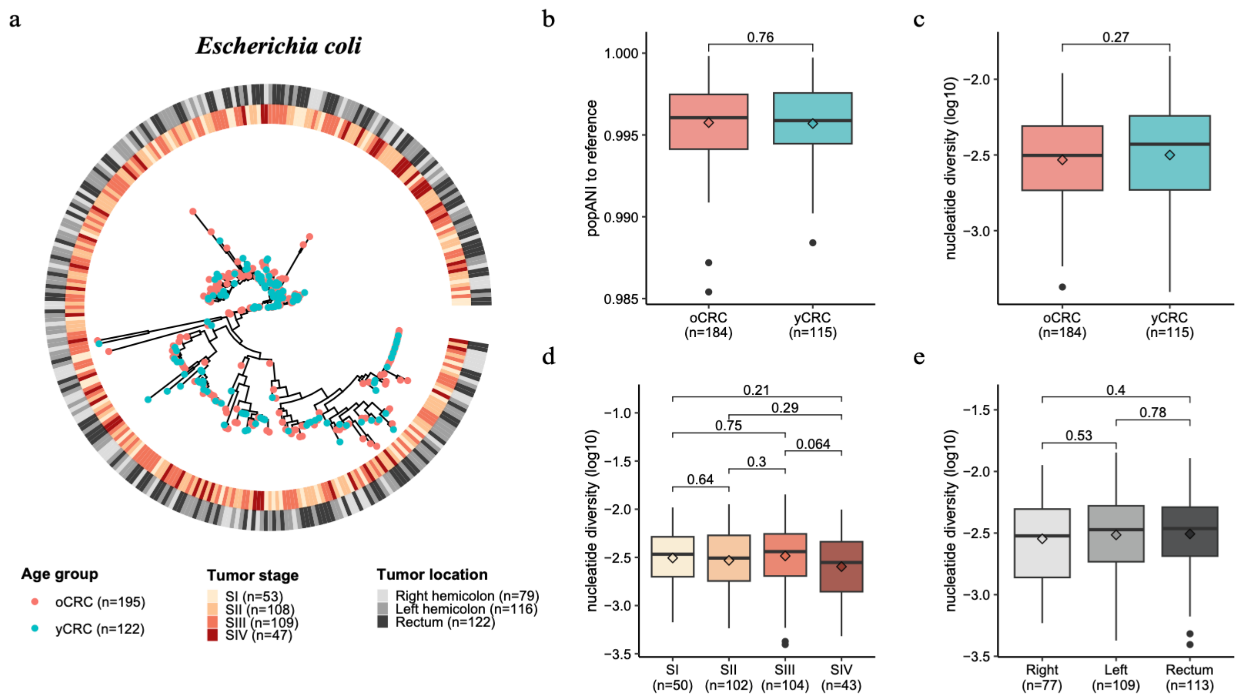

Supplementary Figure 7: Phylogenetic and genomic analysis of *Escherichia coli* strain in CRC. (**a**) Phylogenetic tree of *E. coli* constructed based on 24 marker genes. Tips are samples and colored by age group. Rings outside the tree indicate tumor stage and location. Only samples with reads mapped to at least 20 marker genes are displayed. (**b**) Population average nucleotide identity (popANI) values to reference genome (RefSeq GCF_003697165.2). (**c**), (**d**) and (**e**) are values of genome-wide nucleotide diversity stratified by age, tumor stage and location. Only panel (**a**) samples with reads mapped to the reference genome reaching a genome-wide breadth >0.1 and coverage >0.2 were shown. Boxplot conventions are consistent with the description in **Supplementary Figure 1**.

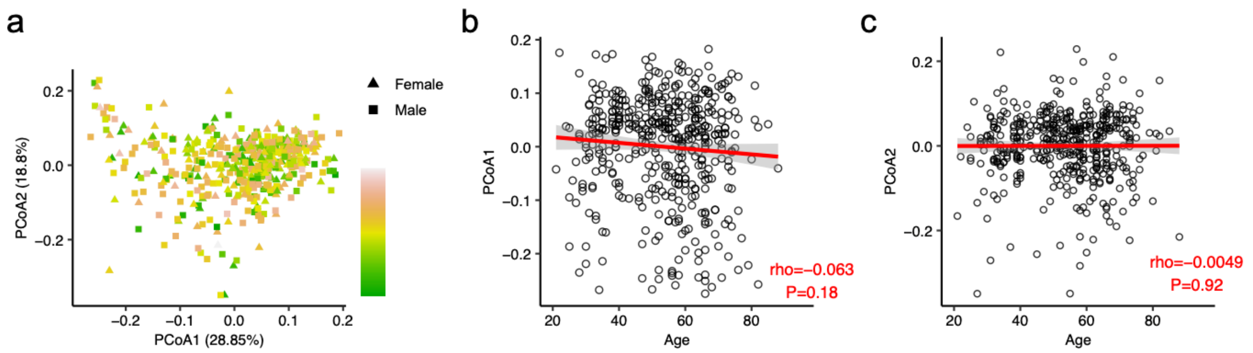

Supplementary Figure 8. Overall distribution of the Guangzhou samples based on microbial pathway profile. (**a**) Two-dimension scatter plot shows the overall pattern of the Guangzhou samples (n=460). Principle coordinate analysis (PCoA) was performed based on the Bray-Curtis distance. Each point represents one sample and color scale indicates age. Samples from female and male patients are in triangles and squares, respectively. Scatterplot of relationship between age and PCoA axis 1 (**b**) and PCoA axis 2 (**c**). The solid red line was fitted by smooth function in R and the grey area is the 95% confidence interval.

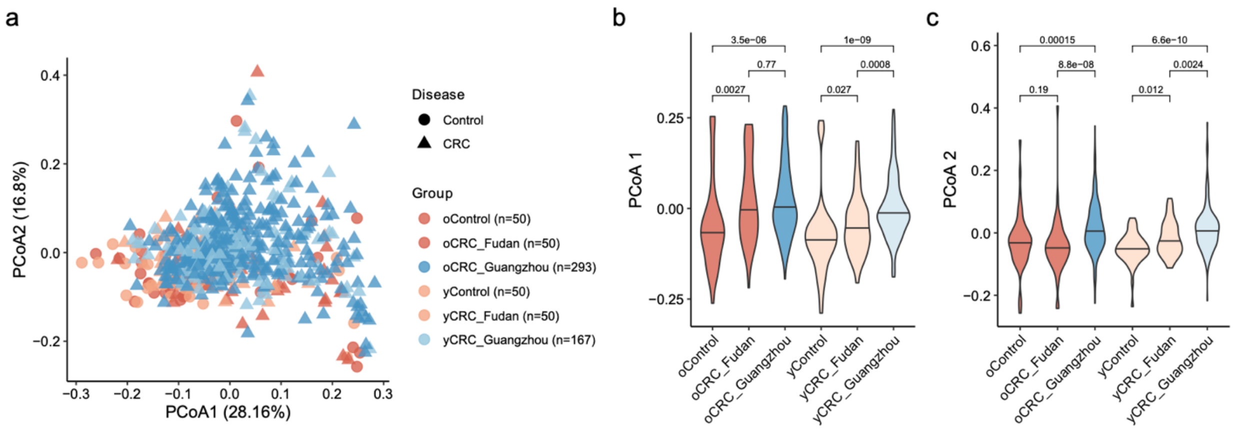

Supplementary Figure 9. Overall distribution of the Guangzhou and Fudan samples based on microbial pathway profile. (**a**) Two-dimension scatter plot shows the overall distribution of Fudan and Guangzhou samples. Principle coordinate analysis (PCoA) was performed based on the Bray-Curtis distance. Each point represents one sample. Samples from Fudan and Guangzhou cohorts are in red and blue, respectively. Circles are control samples, while triangles are CRC samples. Violin plots show values of PCoA axis 1 (**b**), PCoA axis 2 (**c**). P values on the top were calculated by two-side Wilcoxon rank-sum test. The thick horizon line indicates the 50% percentile.

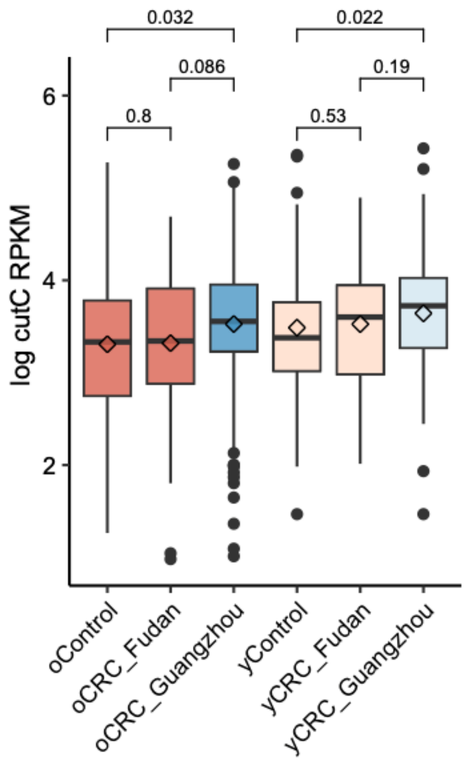

Supplementary Figure 10. Abundance distribution of the well-known CRC-enriched microbial *cutC* gene. The y-axis shows the number of mapped reads per kilobase per million reads (RPKM) in log scale. P values on the top were calculated by two-side Wilcoxon rank-sum test. Boxplot conventions are consistent with the description in **Supplementary Figure 1**.

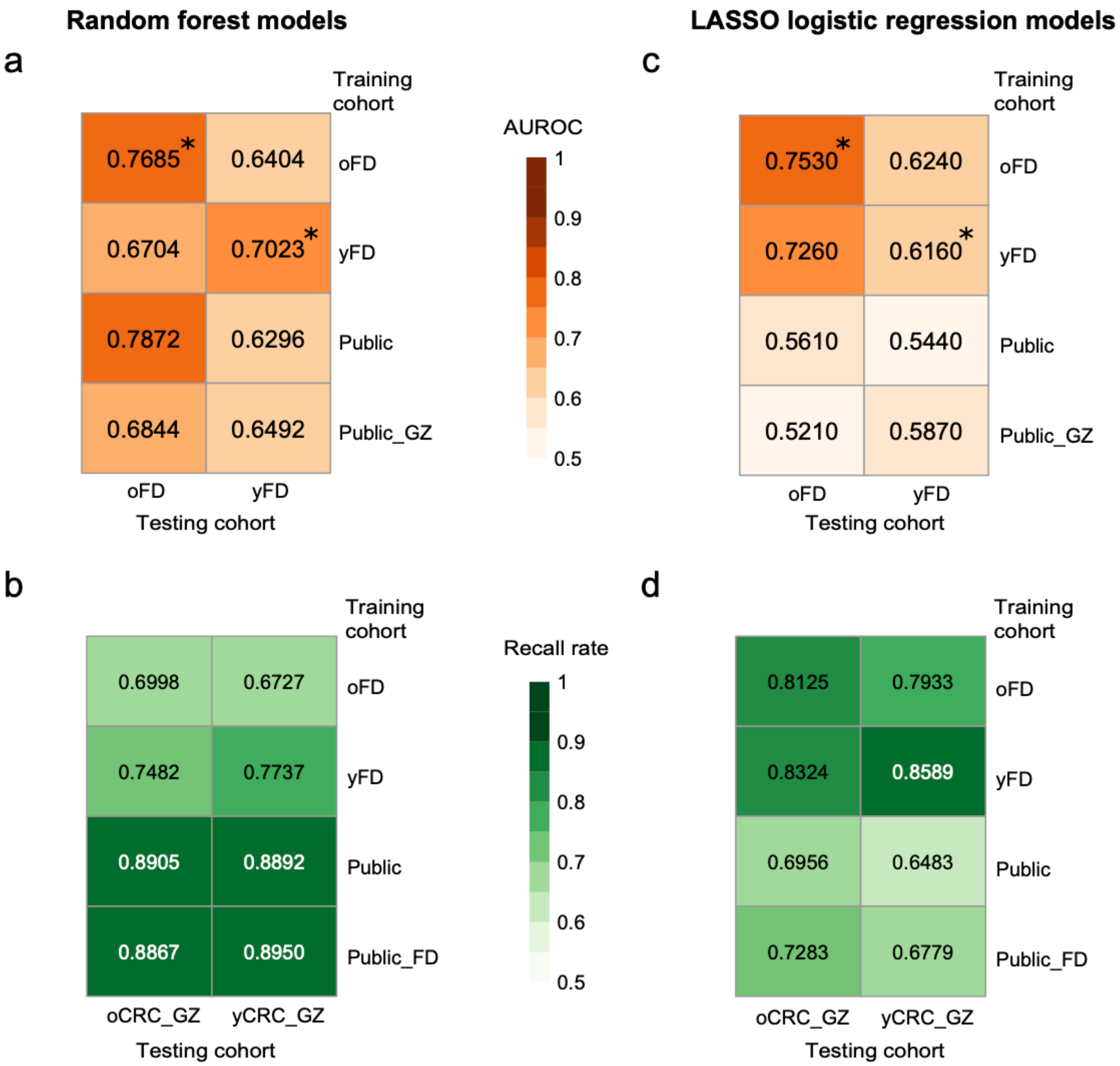

Supplementary Figure 11. Prediction performance of microbial pathway-based classification models. Prediction performance on oCRC and yCRC in Fudan and Guangzhou cohorts for models trained on pathway-level abundances from different datasets. Models were trained on two different methods: random forest and LASSO logistic regression. The numbers are the area under receiver operator curve (AUROC) for (**a** & **c**), and recall rate for (**b** & **d**). Asterisks denote values averaged over 100 times ten-fold cross-validation. Abbreviations: oFD means the 100 metagenomes of Fudan oCRC and oControl; yFD means the 100 metagenomes of Fudan yCRC and yControl; Public means 1,262 public metagenomes; Public_GZ means 1,262 public metagenomes plus 460 Guangzhou metagenomes; Public_FD means 1,262 public metagenomes plus 200 Fudan metagenomes.

Supplementary Table 1. Age distribution of the publicly available CRC metagenomic dataset. The table includes ten cohorts from eight previous studies. The Fudan study (Yang *et al*. 2021) aimed to investigate the CRC microbiome in young-onset patients was not included.

**Cohort**	**CRC+ Control**	**CRC**			**Control**		
		**all**	**age<50**	**age<40**	**all**	**age<50**	**age<40**
FengQ_2015	107	46	4	0	61	3	0
GuptaA_2019	60	30	4	0	30	15	15
ThomasAM_2019_a	46	25	0	0	21	0	0
ThomasAM_2019_b	60	32	4	2	28	4	0
ThomasAM_2019_c	80	40	10	4	40	9	0
VogtmannE_2016	104	52	8	3	52	11	2
WirbelJ_2018	125	60	7	2	65	17	8
YachidaS_2019	438	187	28	5	251	52	16
YuJ_2015	128	75	4	1	53	0	0
ZellerG_2014	114	53	3	0	61	8	5
**Sum**	**1262**	**600**	**72**	**17**	**662**	**119**	**46**
