# Supplementary material for: Consistent signatures in the human gut microbiome of old- and young-onset colorectal cancer
Source: Nat Commun. 2024 Apr 22;15:3396. doi: 10.1038/s41467-024-47523-x (PMC11035630; doi:10.1038/s41467-024-47523-x)
Supplement: Supplementary file 3 — Description_of_Additional_Supplementary_Files [file 41467_2024_47523_MOESM3_ESM.pdf]

Description of Additional Supplementary Files

Supplementary Data 1. Summary information of metadata for the Guangzhou and Fudan cohorts. Age bins means [20,30), [30,40), [40,50), [50,60), [60,70) and ≥70. The information for the Fudan cohort was derived from Yang et al. P values were calculated by chi-square test for MMR, MSH2, MSH6, MLH1, PMS2, HER2 and BRAF status. Fisher test was used if any count was less than 5. P values for BMI was calculated by two-side Wilcoxon–Mann–Whitney for two group comparison and Pearson correlation for multiple groups comparison.

Supplementary Data 2. Weighted and unweighted associations between species abundance and age. The table shows the associations with and without the adjustment of covariates. The associations were calculated by weighted (relative abundance) and unweighted (presence and absence) methods. Only species with prevalence rate no less than 15% were shown. P values were calculated by Spearman correlation for weighted analysis and MaAsLin2 for unweighted analysis.

Supplementary Data 3. Two-side Wilcoxon rank-sum test results of species abundance between old and young patient groups. Alteration direction was determined by median abundance. In the case of equal median abundance, mean abundance was used.

Supplementary Data 4. Two-side Wilcoxon rank-sum test results of species abundance between CRC and control groups. Alteration direction was determined by median abundance. In the case of equal median abundance, mean abundance was used.

Supplementary Data 5. Weighted and unweighted associations between microbial pathway abundance and age. The table shows the associations with and without the adjustment of covariates. The associations were calculated by weighted (relative abundance) and unweighted (presence and absence) methods. Only pathways with prevalence rate no less than 15% were shown. P values were calculated by Spearman correlation for weighted analysis and MaAsLin2 for unweighted analysis.

Supplementary Data 6. Two-side Wilcoxon rank-sum test results of microbial pathway abundance across different groups. Alteration direction was determined by median abundance. In case of equal median abundance, mean abundance was used.

Supplementary Data 7. Two-side Wilcoxon rank-sum test results of microbial *cutC* gene abundance across different groups. Only 65 gene ortholog present in at least one sample of each group were shown. Alteration direction was determined by median abundance. In case of equal median abundance, mean abundance was used. The abundance means reads per kilobase per million (RPKM).

Supplementary Data 8. Weighted and unweighted associations between microbial taxonomic markers and tumor characteristics. The table shows the associations with and without the adjustment of covariates. The associations were calculated by weighted (relative abundance) and unweighted (presence and absence) methods. Only 18 taxa reported in **Figure 2, S1, S2 and S3** were shown. P values were calculated by Spearman correlation for weighted analysis and MaAsLin2 for unweighted analysis.

Supplementary Data 9. Summary of quality control for shotgun metagenomic sequencing reads of the Guangzhou cohort.

Supplementary Data 10. CRC associated taxa downloaded from the gutMDisorder database. The table was downloaded in March 2023.
