# Supplementary material for: Consistent signatures in the human gut microbiome of old- and young-onset colorectal cancer
Source: Nat Commun. 2024 Apr 22;15:3396. doi: 10.1038/s41467-024-47523-x (PMC11035630; doi:10.1038/s41467-024-47523-x)
Supplement: Supplementary file 14 — Reporting Summary [file 41467_2024_47523_MOESM14_ESM.pdf]

Corresponding author(s): Dr Youwen Qin
Prof Pei-Rong Ding

Last updated by author(s): Mar 20, 2024

Reporting Summary

Nature Portfolio wishes to improve the reproducibility of the work that we publish. This form provides structure for consistency and transparency in reporting. For further information on Nature Portfolio policies, see our Editorial Policies and the Editorial Policy Checklist.

Statistics

For all statistical analyses, confirm that the following items are present in the figure legend, table legend, main text, or Methods section.

n/a	Confirmed	
☐	☒	The exact sample size (*n*) for each experimental group/condition, given as a discrete number and unit of measurement
☐	☒	A statement on whether measurements were taken from distinct samples or whether the same sample was measured repeatedly
☐	☒	The statistical test(s) used AND whether they are one- or two-sided<br>*Only common tests should be described solely by name; describe more complex techniques in the Methods section.*
☐	☒	A description of all covariates tested
☐	☒	A description of any assumptions or corrections, such as tests of normality and adjustment for multiple comparisons
☐	☒	A full description of the statistical parameters including central tendency (e.g. means) or other basic estimates (e.g. regression coefficient) AND variation (e.g. standard deviation) or associated estimates of uncertainty (e.g. confidence intervals)
☐	☒	For null hypothesis testing, the test statistic (e.g. *F*, *t*, *r*) with confidence intervals, effect sizes, degrees of freedom and *P* value noted<br>*Give P values as exact values whenever suitable.*
☒	☐	For Bayesian analysis, information on the choice of priors and Markov chain Monte Carlo settings
☒	☐	For hierarchical and complex designs, identification of the appropriate level for tests and full reporting of outcomes
☐	☒	Estimates of effect sizes (e.g. Cohen's *d*, Pearson's *r*), indicating how they were calculated

Our web collection on statistics for biologists contains articles on many of the points above.

Software and code

Policy information about availability of computer code

Data collection	No particular software was used for data collection.
Data analysis	All software used for data analyses is publicly available and is cited in the Methods section where possible. These software are:<br>command-line tools: metapi workflow (https://github.com/ohmeta/metapi/), bowtie2 v2.4.2 (https://github.com/BenLangmead/bowtie2), MetaPhlAn 3 (https://github.com/biobakery/MetaPhlAn/wiki/StrainPhlAn-3), HUMAnN 3 (https://github.com/biobakery/biobakery/wiki/humann3), StrainPhlAn 3 (https://github.com/biobakery/MetaPhlAn/wiki/StrainPhlAn-3), megahit v1.2.9 (https://github.com/voutcn/megahit), GTDB-Tk v2.1.0 (https://github.com/Ecogenomics/GTDBTk), dRep v3.2.0 (https://github.com/MrOlm/drep), inStrain v1.8.0 (https://instrain.readthedocs.io/en/latest/)<br>R v4.3.1: vegan v2.6.4, mlr3 v0.17.2, SIAMCAT v2.6.0, ANCOMBC2 v2.4.0, MaAsLin2 v1.16.0<br>Code to replicate our microbiome-based CRC prediction is deposited at https://github.com/Owen-haha/CRCmicrobiome

For manuscripts utilizing custom algorithms or software that are central to the research but not yet described in published literature, software must be made available to editors and reviewers. We strongly encourage code deposition in a community repository (e.g. GitHub). See the Nature Portfolio guidelines for submitting code & software for further information.

Data

Policy information about availability of data

All manuscripts must include a data availability statement. This statement should provide the following information, where applicable:

- Accession codes, unique identifiers, or web links for publicly available datasets
- A description of any restrictions on data availability
- For clinical datasets or third party data, please ensure that the statement adheres to our policy

The data (including fastq sequences and assembly results) that support the findings of this study have been deposited into CNGB Sequence Archive (CNSA) of China National GeneBank DataBase (CNGBdb) with accession number CNP0004314. A copy of data has also been deposited into Genome Sequence Archive (GSA, https://ngdc.cncb.ac.cn/gsa/) with submission number of HRA006629.
The public data from Yang et al, 2021 was downloaded from the NIH National Center for Biotechnology Information Sequence Read Archive (SRA) with BioProject ID PRJNA763023 (https://www.ncbi.nlm.nih.gov/bioproject/PRJNA763023).
The public data published before 2020 was downloaded from Beghini et al, 2021 (https://elifesciences.org/articles/65088#data).

Research involving human participants, their data, or biological material

Policy information about studies with human participants or human data. See also policy information about sex, gender (identity/presentation), and sexual orientation and race, ethnicity and racism.

Reporting on sex and gender	The self-reported sex (biological attribute) was used in this study. The proportion of male and female (biological attribute) is included in Supplementary Table 2. Some sex-related analysis was performed in this study.
Reporting on race, ethnicity, or other socially relevant groupings	Race / ethnicity was not recorded or analyzed in this study.
Population characteristics	We recruited 460 CRC patients from a single hospital in Guangzhou (Methods). All patients were treatment naïve by the time of enrollment. Our cohort included patients with a wide age range, from 21 to 88 years old (Figure 1A), with 95 patients diagnosed under the age of 40 and 167 patients under the age of 50. Across all age groups, there were more male patients than female patients. 14.8% (n=68) of cancers were stage I, 32.0% (n=147) stage II, 36.1% (n=166) stage III and 17.2% (n=79) stage IV; 24.8% (n=114) were from the right hemicolon, 34.78% (n=160) left hemicolon and 40.43% (n=186) rectum.
Recruitment	All patients were recruited from Sun Yat-sen University Cancer Center in Guangzhou. The inclusion criteria were newly diagnosed pathologically proven locally advanced rectal adenocarcinoma. Patients with any of previous tumor history or tumor treatment history were removed from the study. Medical practitioners interviewed patients and reviewed their eligibility, willingness to participate and sign the informed consent. Patient geography and knowledge of the study may have biased study participation.
Ethics oversight	All patients were recruited from Sun Yat-sen University Cancer Center in Guangzhou in accordance with the study protocol approved by the Ethics Committee of Sun Yat-sen University Cancer Center (B2019-214-X02). Informed consent was obtained from every patient.

Note that full information on the approval of the study protocol must also be provided in the manuscript.

Field-specific reporting

Please select the one below that is the best fit for your research. If you are not sure, read the appropriate sections before making your selection.

☒ Life sciences ☐ Behavioural & social sciences ☐ Ecological, evolutionary & environmental sciences

For a reference copy of the document with all sections, see nature.com/documents/nr-reporting-summary-flat.pdf

Life sciences study design

All studies must disclose on these points even when the disclosure is negative.

Sample size	The primary objective of this study was to characterize the gut microbiota of young-onset colorectal cancer. At the time of its design there was limited data to estimate the required sample size. Therefore, no statistical sample size or power calculation could be performed a priori.
Data exclusions	No data was excluded intentionally. Samples from all 460 individuals were included in our analysis. Sensitivity analysis was performed on 372 individuals from the Guangzhou cohort who had no missing data for any covariate.
Replication	No technical replicates of metagenomic sequencing was performed. Findings were validated in an independent cohort from a recent published study (Yang et al. 2021).
Randomization	This is an observational cohort study. The study does not involve an intervention, so patients were not randomized.

Blinding	The sample testing staff were blinded to all clinical information. This is an observational cohort study. The study does not involve an intervention, so blinding was not required in other aspect.

Reporting for specific materials, systems and methods

We require information from authors about some types of materials, experimental systems and methods used in many studies. Here, indicate whether each material, system or method listed is relevant to your study. If you are not sure if a list item applies to your research, read the appropriate section before selecting a response.

Materials & experimental systems

n/a	Involved in the study
☒	☐ Antibodies
☒	☐ Eukaryotic cell lines
☒	☐ Palaeontology and archaeology
☒	☐ Animals and other organisms
☒	☐ Clinical data
☒	☐ Dual use research of concern
☒	☐ Plants

Methods

n/a	Involved in the study
☒	☐ ChIP-seq
☒	☐ Flow cytometry
☒	☐ MRI-based neuroimaging
